# A rare case of a simultaneous post-dissection saccular aneurysm of the ascending aorta and large pulmonary artery aneurysm with secondary embolism: a case report

**DOI:** 10.1186/s13019-024-02865-x

**Published:** 2024-06-21

**Authors:** S. R. van Dinter, T. Arslan, S. Boerman, F. N. Hofman, P. Klein

**Affiliations:** 1 Department of Cardiothoracic Surgery, Radboud University Medical Center, Geert Grooteplein Zuid 10, Nijmegen, 6525 GA The Netherlands; 2 Department of Cardiothoracic Surgery, St. Antoniushospital, Nieuwegein, The Netherlands; 3 Department of General Surgery, University Hospital Leuven, Leuven, Belgium; 4 Department of Pulmonary Medicine, St. Antoniushospital, Nieuwegein, The Netherlands; 5 Department of Cardiothoracic Surgery, Amsterdam Medical Center, Amsterdam, The Netherlands

**Keywords:** Aneurysm, Ascending aorta, Pulmonary artery, Dissection, Embolism, Pulmonary arterial hypertension, Hypertension

## Abstract

**Background:**

Aneurysms of the pulmonary arteries and the ascending aorta are rare, and both bear a high mortality risk if left untreated. In general, these entities are primarily caused by etiologies such as hypertension, pulmonary arterial hypertension, infection or congenital disorders. Treatment requires a rapid diagnostic work-up or even immediate surgical intervention in acute cases. Nevertheless, surgery entails serious perioperative risks, in particular in patients with multiple comorbidities.

**Case presentation:**

We discuss a 70-year-old woman presented with decompensated heart failure based on severe pulmonary artery hypertension, coincided by a massive pulmonary artery aneurysm with secondary embolism. Additional diagnostic imaging also showed a chronic post-dissection, saccular aneurysm of the ascending aorta. To our knowledge, this simultaneous diagnosis of a saccular aneurysm of the ascending aorta and a large aneurysm of the pulmonary artery with secondary embolism has not yet been described. Nonetheless, conservative treatment was chosen due to extensive pulmonal and cardiovascular comorbidities and the high-risk profile of surgery.

**Conclusions:**

Extensive aneurysmatic disease of the pulmonary arteries and ascending aorta come with a serious burden of disease, especially if coincided by severe pulmonal and cardiovascular comorbidities. Both conditions can be curatively treated by surgical intervention. However, in every case the risk of surgery and the patient’s vitality, comorbidities and wishes should be taken into account to formulate an adequate treatment plan. Therefore, shared decision making is of utter importance.

## Background

Pulmonary artery aneurysm (PAA) is a rare entity and poses a serious risk of rupture or dissection when exposed to high tension. Various etiologies for PAA have been described, differentiating between a high-pressure and low-pressure PAA. In high-pressure PAA, arterial dilatation is primarily caused by high vessel wall tension, such as in pulmonary arterial hypertension (PAH), while a low-pressure PAA can be caused by a broad spectrum of causes including infection, vasculitis, structural/congenital cardiovascular diseases, and atherosclerosis [1, 2]. Coexistence of PAA with a pulmonary embolism (PE) has also been described in both high-pressure etiologies like chronic thromboembolism pulmonary hypertension (CTEPH) as low-pressure etiologies such as in Behcet’s disease and Hughes-Stovin syndrome [1]. We report on a case of large PAA of the pulmonary trunk and main pulmonary arteries secondary to PAH, coincided by the diagnosis of secondary thromboembolism of the left PAA and a post-dissection saccular aneurysm of the ascending aorta (TAA). To our knowledge, a few cases have been reported on the simultaneous occurrence of PAA, PE and TAA [3, 4]. However, the combination of a large PAA of this caliber, with secondary PE and a saccular TAA of the ascending aorta has not yet been described.

## Case presentation

A 70-year-old woman was referred by the general practitioner to the outpatient clinic of pulmonology due to an abnormal perihilar mediastinal mass on chest X-ray. She complained of fatigue, malaise, and progressive weight loss. Presented was a frail woman, having lost 20 kg in 2 years, frequently needed additional sleep and experienced dyspnea since a COVID-19 infection in 2022. Despite actively smoking (40 packyears), she had never experienced pulmonary complaints. Her medical history included arterial hypertension, ischemic stroke leading to reduced mobility and chronic neuropathic pain, overactive bladder syndrome with chronic cystitis. Her surgical history included hysterectomy and unilateral adnexectomy, laparoscopic left-sided adrenalectomy due to a hyperplastic aldosteron-producing adrenal gland, later followed by bilateral renal angioplasty/stenting due to fibromuscular dysplasia and resection of a left-sided adrenal cyst.

**Fig. 1:**
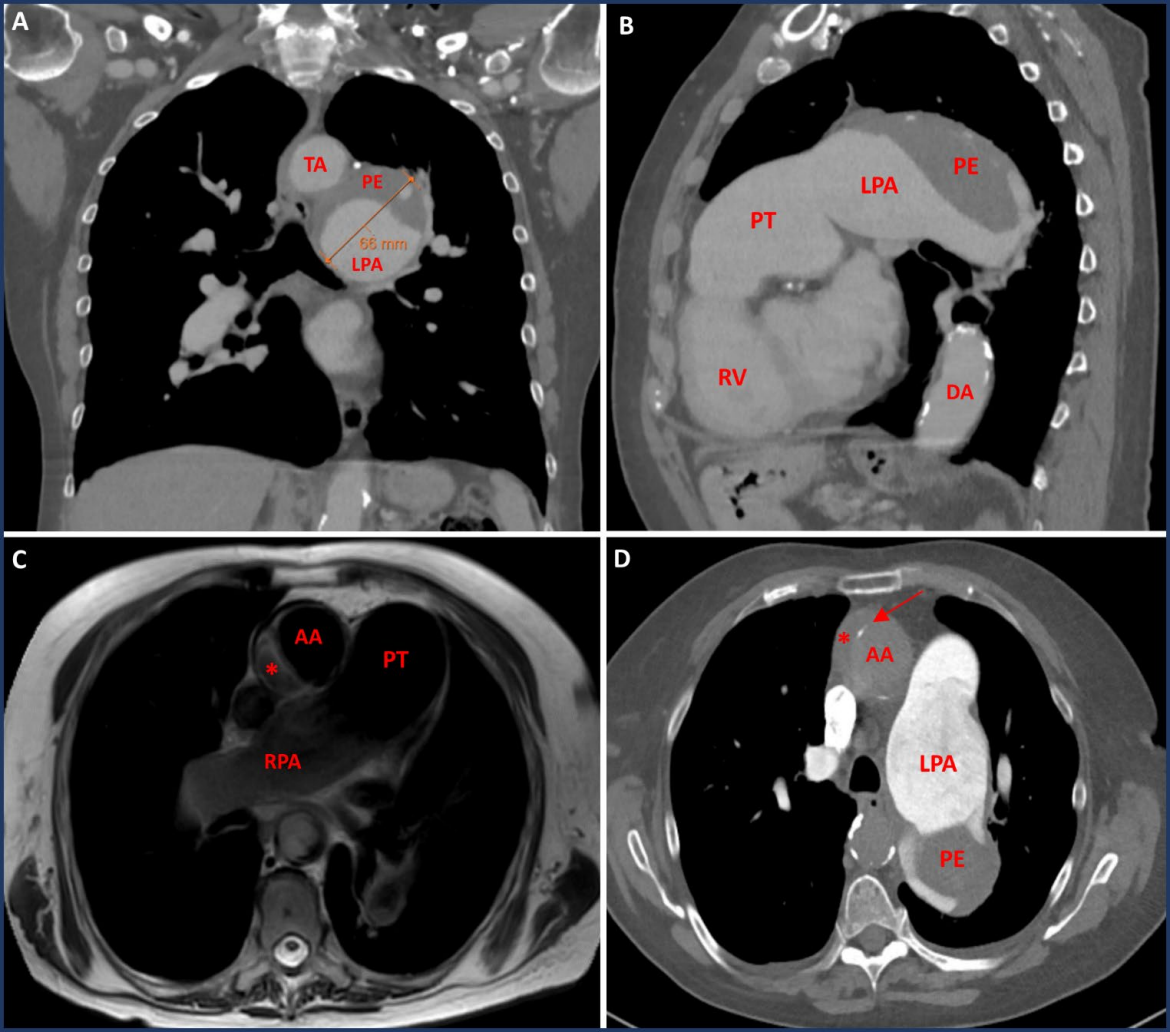
Diagnostic work-up imaging with CMR and CT scan of the PAA and PE. Initial CT and CMR scan in the diagnostic work-up regarding the PAA and PE. Figure 1A and B: CT scan with frontal and sagittal view of the transverse aortic arch and left pulmonary artery with pulmonary embolism. Figure 1C and D: CMR and CT scan with transversal view of the aneurysmatic pulmonary trunk, right and left pulmonary artery, as well as the ascending aorta with retrospectively diagnosed saccular post-dissection aneurysm (depicted by *). The arrow indicates calcifications marking the aortic lumen AA = ascending aorta, DA = descending aorta, LPA = left pulmonary artery, PE = pulmonary embolism, PT = pulmonary trunk, RPA = right pulmonary artery, RV = right ventricle, TA = transverse aortic arch

Physical examination was without abnormalities and lab examination only showed mild renal insufficiency. Pulmonary function test revealed chronic obstructive pulmonary disease (COPD) GOLD-class III with a diffusion capacity of 35%. Subsequent computed tomography (CT) scan showed bilaterally dilated pulmonary arteries with a left pulmonary artery aneurysm of 6.6 cm, which was also affected by the presence of a mural thrombus (Fig. 1A&B). The latter showed no pathological FDG uptake on PET-CT. A transthoracic echocardiogram (TTE) indicated signs of right ventricular (RV) pressure overload, including a dilated, hypertrophic RV, a dilated right atrium, and moderate tricuspid valve insufficiency. Estimated pulmonary artery pressure (PAP) was 75/21 mmHg. A cardiovascular magnetic resonance (CMR) scan was performed, and lack of contrast uptake suggested that the intra-arterial mass was likely a thrombus rather than an angiosarcoma. Due to clinical aggravation into RV failure, the patient was hospitalized for treatment with iv diuretics. Additionally, therapeutic oral anticoagulation and COPD treatment were initiated.

One month after treatment, estimated RV systolic pressure on follow-up TTE was 67 + 8 mmHg and a moderate-severe tricuspid regurgitation was observed. A right heart catheterization showed a mean PAP of 49 mmHg, mean right atrium pressure of 16 mmHg, pulmonary artery wedge pressure of 10 mmHg, cardiac output of 2.9 l/min with corresponding pulmonary vascular resistance of 13.4 WU, indicating severe precapillary PAH (WHO group 1). Ventilation/perfusion scan showed bilateral matched (sub)segmental perfusion and ventilation defects. Additional examination, including autoimmune serology, did not reveal a cause of PAH. The diagnosis of idiopathic precapillary PAH was made, and Riociguat® and Macitentan® were initiated while continuing diuretics and anticoagulation. At that time, surgical thrombectomy and vascular reconstruction were considered. However, because of the compromised pulmonary hemodynamics, patients’ comorbidities and significant perioperative risks, non-surgical therapy for optimalisation of pulmonary hemodynamics was preferred.

**Fig. 2:**
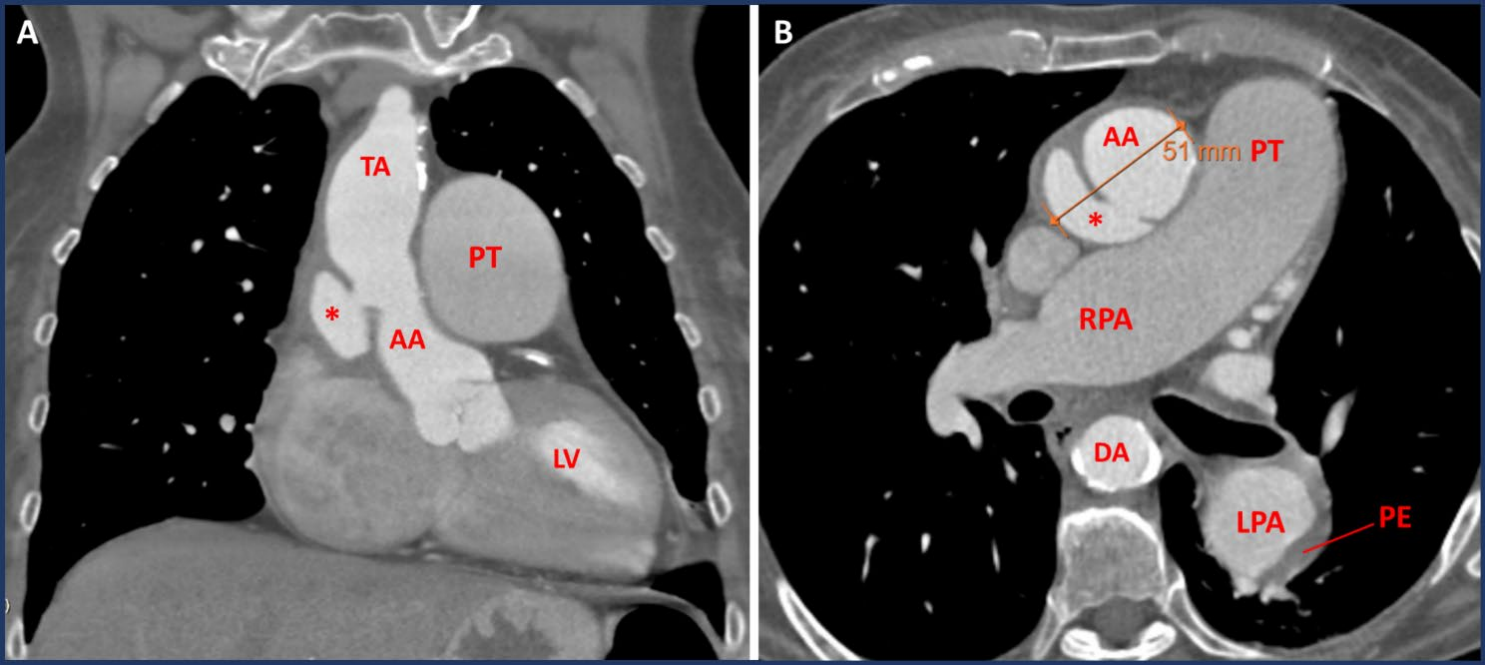
Additional CT angiography of the post-dissection aneurysm. A CT angiography scan of the ascending thoracic aorta, showing the saccular post-dissection aneurysm (depicted by *) at the right-sided posterior section of the mid-ascending aorta in coronal (Fig. 2A) and transversal (Fig. 2B) view AA = ascending aorta; DA = descending aorta; LPA = left pulmonary artery; LV = left ventricle; PE = pulmonary embolism; PT = pulmonary trunk; RPA = right pulmonary artery; TA = transverse aortic arch

Later, the patient was readmitted for COPD exacerbation and acute-on-chronic renal failure due to gastroenteritis. During this readmission, four months after initial diagnosis, the radiologist noted that a TAA could be suggested retrospectively on the initial CMR and CT-scan (Fig. 1C&D). This post-dissection TAA was initially missed because the CT-scan was mainly focused on the pulmonary anatomy. As a result, with the contrast agent in the wrong imaging phase, spotting the false lumen of the dissection can be difficult. Nevertheless, diagnosis of the TAA on the initial scan had been possible if the fine calcifications of the inner aortic layer had been noticed. A subsequent CT angiography of the aorta confirmed a supracoronary saccular aneurysm (40 × 40 × 15 mm) on the right-posterior side of the ascending aorta with a maximum diameter of 51 mm (Fig. 2). On further anamnesis, the patient recalled experiencing interscapular pain a few months before initial admission. Therefore, the aneurysm was suspected to be a post-dissection saccular aneurysm. Apart from the patient declining surgical intervention, surgery was deemed too high risk considering the extensive comorbidities, frailty and high-risk profile of surgery.

All things considered the patient was initially doing fairly well. Unfortunately, approximately one year after presentation, she passed away due to severe COPD exacerbation.

## Discussion

PAA is a rare diagnosis with an incidence of 1:14000, and co-occurrence of PE and aortic dissection is rather exceptional [1]. Considering the reported prevalence of 0.6% of all saccular aortic aneurysms occurring in the ascending aorta, simultaneous diagnosis of PAA with PAH and secondary PE, and a saccular, post-dissection TAA of the ascending aorta has not yet been described [3, 5].

Multiple risk factors and causes can contribute to TAA and PAA development. Chronic arterial hypertension has been described as the primary risk factor for TAA [6]. The patient we presented had long-standing arterial hypertension due to renal fibromuscular dysplasia. Regarding the etiology of the PAA, both COPD and PE have been described as risk factor for developing PAH and consequently high-pressure PAA [1]. Nevertheless, from our perspective, the severity of PAH cannot be solely explained by the COPD or PE, especially since no other thrombi were visible on CT and the ventilation/perfusion scan lacked large perfusion defects. Notably, COVID-19 appears to mark the onset of clinical deterioration in our case. Patients infected by COVID-19 have been reported to have an adjusted risk ratio of 34.7 to acquire PE [7]. Therefore, this could have initiated the embolization process in the low-flow PAA. Even though no cause of the PAH could be identified, the comorbidities mentioned above may have accelerated time to clinical worsening.

Surgery remains the gold standard for the curative treatment of a TAA of the ascending aorta, as well as PAA and CTEPH. Open techniques for PAA range from aneurysmorrhaphy or aneurysmectomy to a lobectomy or pneumonectomy, and in case of CTEPH a pulmonary thromboendarterectomy can be performed. Endovascular options to treat PAA have been described, although these options are only feasible for localized and small saccular aneurysms [1]. Regarding this TAA of the ascending aorta, open repair with a supracoronary ascending aorta replacement (SCAR) is the most commonly used technique. In high-risk patients there have been, albeit still in its infancy, several reports of endovascular and hybrid approaches with branched, double-barrel, chimney, snorkel, and dissection stent grafts [5]. Nonetheless, a zone zero thoracic endovascular aortic repair (TEVAR) requires an adequate proximal and distal landing zone, which in the presented case was of minimal length and thereby entailing a risk to compromise the coronary ostia.

Prognoses of the mentioned diseases vary, depending on the clinical phase and chosen therapy. Considering a Stanford type A acute aortic dissection, close to 75% of the elderly patients die within 2 weeks after clinical presentation without surgical treatment. Operative mortality - depending on clinical presentation and co-morbidities is around 20%. Five-year survival rates vary between 32% and 100% [6, 8]. Contrastingly, dependent on the aortic diameter, survival of chronic TAA is preferable. Kim et al. report a predicted 5-year adverse aortic events rate (rupture, emergency aortic surgery or sudden death) of 11.4% for a 70-year-old patient with a 50 mm chronic TAA with conservative treatment, compared to a 5-year survival of 87% after surgery [9]. As for high-pressure PAA, mortality can range from 50 to 100% in the event of rupture or dissection [1]. Nonetheless, a review by Duijnhouwer et al. describes only 0.2% of confirmed PAA dissection, alongside 2.7% of possible PAA dissection/rupture in patients with unexpected death yet with lacking autopsy results [2]. For this reason, their advice is a conservative strategy if the PAA has a diameter < 75 mm or a growth rate > 2 mm/year. Yet, it is recommended to consider surgical treatment if the PAA is exposed to an absolute pressure of > 50 mmHg or if the aneurysmatic anatomy leads to thrombus formation, which both were the case in our presented patient [2, 10].

In the described case, open surgery was the most feasible option as curative care. We considered a pulmonary endarterectomy with resection of pulmonary aneurysmatic vasculature and replacement with a vascular prosthesis. This procedure could have been well combined with a SCAR for the post-dissection TAA with aortic clamping just at the proximal aortic arch [1, 2, 5]. . We saw no indication for any additional lung resection as this would not resolve the PAA, and lung transplantation was deemed unfeasible with the patient’s age of 70 and comorbidities [11]. Nonetheless, all of the abovementioned procedures necessitate cardiopulmonary bypass and circulatory arrest, entailing high perioperative risks, particularly given coexisting severe PHT. A consensus statement by the International Society for Heart and Lung Transplantation emphasizes the high risk of narcosis in severe PHT and the necessity of preoperative pulmonary optimalisation, fluid balance correction and maximizing medical therapy. Additionally, severe PHT is correlated with a significantly increased postoperative mortality, reaching 50% mortality in emergency surgery [12]. Considering these survival rates for chronic TAA and PAA, the risks of surgical therapy did not outweigh those of conservative management in our patient.

## Conclusions

The combination of PAA, PE and TAA is a rare finding and comes with a serious burden of disease and high mortality rate, especially if left untreated. Open or endovascular surgical techniques could offer curative options yet come with high perioperative risks. Shared decision making is of utter importance, especially in a complex case like the one presented. All risks including patient’s age, vitality and comorbidities should be weighed against the potential risk reduction for mortality and morbidity and the acquired quality of life. The preference and perspective of the informed patient is leading.

## Data Availability

No datasets were generated or analysed during the current study.

## Abbreviations

CMR: Cardiac Magnetic Resonance
PHT: Pulmonary Hypertension
CT: Computed Tomography
RV: Right Ventricle
CTEPH: Chronic Thromboembolism Pulmonary Hypertension
SCAR: Supracoronary Ascending Aorta replacement
COPD: Chronic Obstructive Pulmonary Disease
TAA: Thoracic Aorta Aneurysm
PAA: Pulmonary Artery Aneurysm
TEVAR: Thoracic Endovascular Aortic Repair
PAP: Pulmonary Artery Pressure
TTE: Transthoracic Echocardiogram
PE: Pulmonary Embolism
